# Endothelial Progenitor Cells Modulate Inflammation-Associated Stroke Vasculome

**DOI:** 10.1007/s12015-019-9873-x

**Published:** 2019-02-09

**Authors:** Sandra A. Acosta, Jea Y. Lee, Hung Nguyen, Yuji Kaneko, Cesar V. Borlongan

**Affiliations:** Department of Neurosurgery and Brain Repair, University of South Florida Morsani College of Medicine, 12901 Bruce B. Downs Blvd., Tampa, FL 33612 USA

**Keywords:** Ischemia, Neuroinflammation, Endothelial progenitor cells, Vasculome, Stem cells

## Abstract

Stroke remains a major unmet clinical need that warrants novel therapies. Following an ischemic insult, the cerebral vasculature secretes inflammatory molecules, creating the stroke vasculome profile. The present study evaluated the therapeutic effects of endothelial cells on the inflammation-associated stroke vasculome. qRT-PCR analysis revealed that specific inflammation-related vasculome genes BRM, IκB, Foxf1, and ITIH-5 significantly upregulated by oxygen glucose deprivation (OGD. Interestingly, co-culture of human endothelial cells (HEN6) with human endothelial cells (EPCs) during OGD significantly blocked the elevations of BRM, IκB, and Foxf1, but not ITIH-5. Next, employing the knockdown/antisense technology, silencing the inflammation-associated stroke vasculome gene, IκB, as opposed to scrambled knockdown, blocked the EPC-mediated protection of HEN6 against OGD. In vivo, stroke animals transplanted with intracerebral human EPCs (300,000 cells) into the striatum and cortex 4 h post ischemic stroke displayed significant behavioral recovery up to 30 days post-transplantation compared to vehicle-treated stroke animals. At 7 days post-transplantation, quantification of the fluorescent staining intensity in the cortex and striatum revealed significant upregulation of the endothelial marker RECA1 and a downregulation of the stroke-associated vasculome BRM, IKB, Foxf1, ITIH-5 and PMCA2 in the ipsilateral side of cortex and striatum of EPC-transplanted stroke animals relative to vehicle-treated stroke animals. Altogether, these results demonstrate that EPCs exert therapeutic effects in experimental stroke possibly by modulating the inflammation-plagued vasculome.

## Introduction

Stroke is the number one cause of disability in the adult population and the fourth leading cause of death in the United States [1–3]. Each year in the United States, an estimated 795,000 people suffer from stroke and therapeutic interventions are limited with only one FDA-approved drug for ischemic stroke, namely tissue plasminogen activator or tPA [4, 5]. Due to its narrow therapeutic window of only 4.5 h after onset, tPA can only be administered to approximately 5% of ischemic stroke patients due to hemorrhagic complications [6, 7]. Therefore, a safe and effective therapeutic intervention is urgently needed for ischemic stroke patients.

Recently, the concept of brain physiology and disease has adopted a more integrative approach whereby different systems, i.e., vascular system and inflammatory system interact and cross-talk [8–11]. These interactions among systems contribute to CNS homeostasis and blood brain barrier (BBB) integrity, which are required for normal brain physiology, and endogenous repair and recovery following brain injury [12–14]. Studies suggest that the blood vessels from the BBB are no longer passive and are actively involved in the brain physiology and pathophysiology [15, 16]. For instance, in healthy CNS, cerebral endothelium is able to provide growth and trophic factors that influence neuroprotection including brain derived neurotrophic factor (BDNF) and fibroblast growth factor (FGF) [16, 17]. Moreover, a pathogenic cerebral endothelium contributes and exacerbates the disease progression by downregulating vasculogenic factors following disease onset i.e., hypertension, diabetes or ischemic stroke [18, 19]. In our laboratory, we have demonstrated that following stroke, distal pathological changes occur indicative of ischemic diaschisis whereby BBB contains “sick” endothelium causing increased autophagosome, degenerated astrocytes and pericytes coupled with peri-vascular edema in brain areas remote from original injury [15]. Recent novel findings revealed that cerebral endothelium can also secrete molecules that may regulate disease processes following ischemic stroke, hereafter referred to as stroke vasculome [20]. This concept of stroke vasculome was recently proposed whereby genes are upregulated uniquely from endothelial cells after the onset of stroke in a mouse model of acute ischemic stroke [20]. Analysis of the stroke vasculome revealed that there is a 72-h upregulation of mixed genes important for blood pressure, vascular contraction, differentiation, tumorigenesis, embryo development, angiogenesis and inflammation [20]. The inflammatory genes in the stroke vasculome include Brahma (BRM), IκB (also called NFκB inhibitor), Foxf1, and ITIH-5.

The upregulation of inflammation-associated genes may be linked to the onset and progression of neuro-inflammation during acute and chronic time points after stroke. Neuro-inflammation is associated with the development of neurodegenerative diseases post stroke injury. At the molecular level, massive inflammatory innate immune responses, hyperactive astrocytes invasion, and altered antigen expression of microglia cells and macrophages contribute to the progressive deterioration of the stroke brain acutely and over time [3, 21]. Previous studies from our laboratory have shown that stem cells are able to release anti-inflammatory cytokines and trophic factors that can regulate and modify the toxic milieu associated with secondary cell death of the ischemic brain [3, 22, 23]. Based on these observations, our long-standing CNS campaign focuses on the concept of stem cell-based therapeutics for stroke, and here advances a novel hypothesis that endothelial progenitor cells (EPCs) directly modulate the inflammation-associated stroke vasculome.

**Fig. 1:**
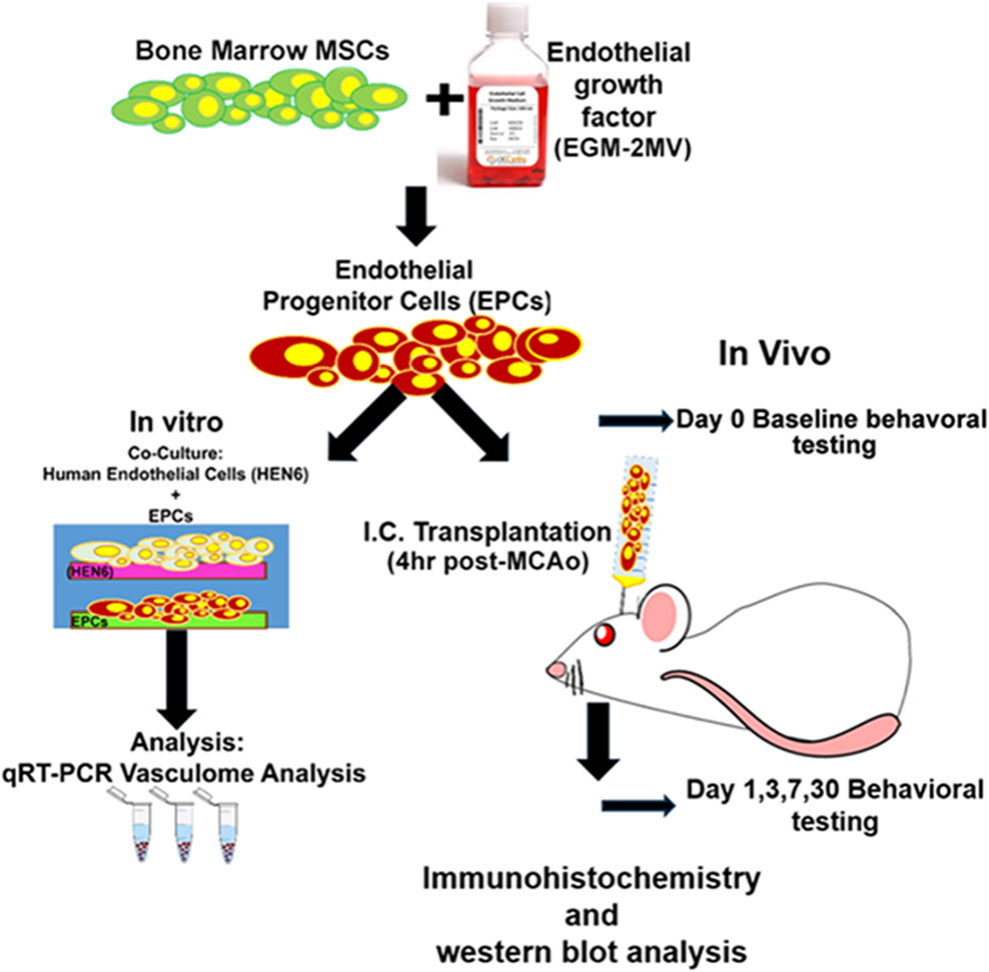
Experimental design. In vitro experiment: Human bone marrow mesenchymal stem cells (BM-MSCs) were seeded with endothelial growth medium (EGM-2MV) supplemented with growth factors. Next, differentiated EPCs were co-culture with human endothelial brain cells (HEN6) and subjected to oxygen and glucose degradation (OGD) and using qRT-PCR RNA vasculome levels were analyzed. In vivo experiment: Motor and neurological tests were performed in all animals on Day 0 before stroke. Animals were subjected to MCAo and reperfused after 1 h. Stroke animals received either vehicle or EPCs at 4 h post MCAo. After motor and cognitive behavioral evaluations, at days 1, 3, 7, animals were euthanized for immunohistochemical and western blot analysis

Transplantation of EPCs is an attractive therapeutic intervention for vascular repair in ischemic stroke because these immature cells (CD4+/VEGFR2+/vWF/CD31+/AC133+) are constantly migrating from the bone marrow to peripheral blood to facilitate endothelium repair/neovascularization during physiological and pathophysiological scenarios via a chemo-attractant signaling pathway, primarily involving a ligand-receptor cross-talk between SDF-1 and CXCR4 [24–31]. Previous studies have shown that exogenous EPC transplantation is able to augment neovascularization at the border of infarcted tissue improving blood vessels formation following stroke [21, 32, 33]. Beyond the vasculogenic effects, and endothelium repair, recent studies have revealed key roles of EPCs on vascular and CNS homeostasis. Transplantation of EPC from Wharton’s jelly of human umbilical cord significantly decreased reactive oxygen species production, and the expression of the inflammatory chemokines macrophage inflammatory-2 and keratinocyte-derived cytokine in an ischemia/reperfusion injury to the kidney [34]. Interestingly, preclinical and clinical studies have demonstrated that EPCs have anti-thrombotic and anti-inflammatory properties via upregulation of cyclooxygenase-2 and downregulation of pro-inflammatory cytokines [35–38]. Moreover, work form our laboratory, using human cerebral endothelial cells (HNE6), demonstrated that transplantation of these cells ameliorated behavioral and histopathological deficits coupled with increased vasculogenesis and neurogenesis in a rat model of ischemic stroke (Fig. 1) [33].

To our knowledge, no study has investigated the potential of EPCs in attenuating the inflammation-associated vasculome following ischemic stroke. Here, we studied the overarching hypothesis that targeting the stroke vasculome via EPC transplantation will dampen the injurious inflammatory response thereby abrogating the progression of this major secondary cell death process in stroke. This study advances the concept of stroke vasculome as an attractive target for developing cell based-therapeutics for neurodegeneration.

## Methods

The protocol for the study has received approval by the Institutional Animal Care and Use Committee. All experiments were conducted in accordance with the United States Public Health Service’s Policy on Humane Care and Use of Laboratory Animals, the National Institute of Health Guide and Use of Laboratory Animals and were approved by the Institutional Animal Care and Use Committee of the University of South Florida, Morsani College of Medicine. Rats were housed 2 per cage in a temperature- and humidity-controlled room that was maintained on 12/12-h light/dark cycles. They had free access to food and water. All necessary steps were performed to minimize animal pain and stress throughout the study. Two-month-old Sprague–Dawley male rats (Envigo, Huntingdon, United Kingdom) served as subjects. All studies were performed by personnel blinded to the treatment condition.

## Cell Culture Procedures

### EPC Differentiation from Human Bone Marrow Mesenchymal Stem Cells

Frozen human bone marrow CD34+ mesenchymal stem cells were purchased from ALLCELLS (Catalog# ABM017F; Alameda, CA). After thawing in a 37 °C water bath, cells were centrifuged at 1500 rpm for 10 min then resuspended with complete endothelial growth medium, EGM-2MV (Lonza, Catalog# CC-3202), containing endothelial cell basal medium-2, 0.5 ml hEGF, 0.5 ml VEGF, 0.5 ml R3-IGF-1, 0.5 ml ascorbic acid, 0.2 ml hydrocortisone, 2.0 ml hFGF-β, 0.5 ml GA and 25.0 ml FBS. 10 million cells per well were seeded on collagen I-coated 48-well plates (Corning, Catalog# 354505) and incubated in 37 °C, 5% CO2 incubator for 7 days for in vivo transplantation and for in vitro immunofluorescence staining. Three different endothelial progenitor cell markers were used including CD133 (1:100, Catalog# NBP2–37741, Novus Biologicals), anti-VEGF (1:100, Catalog# ab2349, Abcam) and anti-von Willebrand factor antibody (1:100, Catalog# MAB3442, Merck Millipore).

### HEN6 Co-culture with EPCs

HEN6 were kindly provided by Dr. Seung Kim (University of British Columbia, Vancouver, BC, Canada). Briefly, primary dissociated cell cultures from the periventricular region of human telencephalic tissues of 14-week gestation were prepared and grown for 10 days. Thereafter, cells were infected with an amphotropic, replication-incompetent retroviral vector containing v-myc. Human endothelial cells (HEN6) were subcultured at ≈90% confluence and subjected to further experiments. Next, HEN6 were phenotypically characterized in vitro and found that these cells were immunocytochemically positive against a human mitochondrial marker (Mito), von Willebrand factor (vWF), and CD31 that the positivity was >99% for each phenotypic marker.

### Preparation of HEN6 with Knockdown of IkB

Using the same cell culture regimen as above but employing the knockdown/antisense technology, the cultured HEN6 ≈ 90% confluence were treated with antisense RNA (IkB or scramble control (Santa Cruz Biotechnology) that was incorporated into the cells for 48 h. HEN6 cells were verified to be deficient of IkB in the appropriate conditions, and no change was observed with the scrambled antisense RNA control (22).

### qRT-PCR

Brains from euthanized rats were instantaneously frozen in liquid nitrogen and stored at −80 °C until processing for quantitative real-time PCR analysis (QRT-PCR). QRT-PCR was performed using the entire brain. Total RNA was extracted from the frozen brain using mirVana™ miRNA isolation kit (Ambion) according to the manufacturer’s instructions and the A260/280 ratio of RNA extraction corresponded to 2.2, which is considered high quality. RNA integrity was confirmed under UV light by visualization of 28S- and 18S-rRNA bands on a denaturing gel containing ethidium bromide**.** For cDNA synthesis, total RNA (2 μg) was reverse-transcribed in a 20-μl volume of reaction mixture, using a RETROscript (Ambion) according to the manufacturer’s instructions. Transcript reactions without the reverse transcriptase enzyme were performed for negative controls in subsequent PCR reactions. For quantitative gene expression, PCR amplification was performed in each reaction mixture containing 300 ng cDNA sample, 200 nM of each primer, 0.1 Unit Taq DNA polymerase (Ambion), 200 μM dNTPs, and 1.5 mM MgCl_2_ (total volume, 25 μl) using mirVana™ qRT-PCR miRNA detection kit (Ambion). The reaction mixture was heated at 94 °C for 3 min, followed by 40 cycles, each consisting of incubation for 30 s at 94 °C, 40 s at 72 °C, and 30 s at 55 °C. Amplification and Sybr Green I (Applied Biosystems) detection were performed on iCycler iQ™ Real-Time PCR Detection System (Bio-Rad). Primers specific for the housekeeping gene glyceraldehyde-3-phosphate dehydrogenase (GAPDH) were used as internal control for the amounts of cDNA generated from each sample. The primer sequences of GAPDH (NW047696) were as follows: forward 5′-ATGGGAAGCTGGTCATCAAC-3′ and reverse 5′-GTGGTTCACACCCATCACAA-3′. For each gene, PCRs were run in triplicate, and the amplified transcripts were quantified using the comparative Ct method. Briefly, threshold cycle (Ct) values were calculated by the equation Δ ΔCt = ΔCt – ΔCt _GAPDH_, where ΔCt is the difference between Ct values and the GAPDH. The relative quantity of mRNA expression in the right hemisphere (i.e., stroke targeted region) was compared with the control hemisphere (i.e., sham operated, intact brain). *Xn* can be calculated by the formula *Xn* = 2^mean^ΔΔ^Ct^. Differences between Ct values were less than 2%. The amplified PCR productions were electrophoresed on a 2% agarose gel containing 0.2 μg/μl ethidium bromide, and the sequences were confirmed using ABI 3730 XL 96-capillary sequencer (data not shown). The productions were visualized under UV light, saved digitally with AlphaImager 2000 (Alpha Innoteck Corporation), and represented as single and theoretical base pair bands. For all experiments, controls without template were incubated. Each primer pair was, when possible, designed to span an exon–exon boundary.

### Stroke and Transplant Surgery

A total of 24 male Sprague-Dawley rats received middle cerebral artery occlusion (MCAo), a well-established stroke model, as described previously [33, 39]. Animals were anesthetized using isoflurane (1.5%–2.5% with oxygen). The skin on the ventral neck was shaved from the jaw to the manubrium and scrubbed with alcohol and chlorhexidine surgical scrub. A skin incision was made over the right common carotid artery. The external carotid was isolated and ligated as far distally as possible. An incision using a pair of microscissors was made in the stump of the external carotid and a 4–0 nylon filament was inserted and passed up into the internal carotid artery until resistance was felt (approximately 15–17 mm), which effectively blocked the middle cerebral artery (MCA). The isoflurane was discontinued and the animals placed in a recovery cage over a warming blanket. We have standardized the MCAo model, with stroke animals showing ≥80% reduction in regional cerebral blood flow (CBF) during the occlusion period as determined by laser Doppler (Perimed). We also found no significant differences in physiological parameters, including PaO2, PaCO2, and plasma pH measurements, in our stroke animals indicating similar degree of stroke insults. Rats that reached the 80% CBF reduction during occlusion were used. After 60 mins of MCAo, animals were re-anesthetized and reperfused by withdrawal of the nylon thread. Finally, animals were placed in a cage over a warming blanket until full recovery from anesthesia. Stroke animals were randomly divided into two groups, stroke-vehicle group and stroke-EPC group. They were tested for behavioral and neurological deficits on Day 0 pre stroke (baseline), and 3 h post stroke. Those animals that showed significant motor and neurological impairments were randomly assigned to receive either EPCs intracerebral transplants targeting the ischemic peri-infarct region 4 h post stroke or vehicle. Animals were fixed to a stereotaxic apparatus (Kopf Instruments). A 26-gauge Hamilton syringe was then lowered into a small burred skull opening (transplant coordinates were adjusted to anterior/posterior (AP): +0.05, and medial/lateral (ML): −0.28, 3 deposits-each of 3 μl at a rate of 3 μl/min for a total of 9 μl in 3 min; striatum: DV -0.5, DV -0.4; Cortex: DV -0.30 [39]. All animals were monitored at days 1, 3, 7, and 30 post-grafting for behavioral and neurological outcomes.

## Behavioral Testing

Each animal was subjected to a series of behavioral tests to analyze motor, and neurological function, pre-stroke, post-stroke and following transplantation. The tests included the elevated body swing test (EBST), forelimb akinesia, paw grasp and rotarod test prior to and after stroke, at days 0, 1, 3, 7, 30.

### EBST Test

EBST is a measure of asymmetrical motor behavior that does not require animal training or drug injection [40]. The rats were held, in the vertical axis, approximately 1 in. from the base of its tail and then elevated to an inch above the surface on which it has been resting. The frequency and direction of the swing behavior were recorded for over 20 tail elevations. A swing was counted when the head of the rat moved more than 10 degrees from the vertical axis to either side. Normally, intact rats display a 50% swing bias, that is, the same number of swings to the left and to the right. A 75% swing bias towards one direction was used as criterion of motor deficit [40]. The total number of swings made to the biased side was added per group and divided by the n, giving us the average number of swings per treatment group.

### Forelimb Akinesia Test

Pre and post stroke surgery, rats from stroke-vehicle and stroke-EPC were evaluated for forelimb akinesia [41]. Ipsilateral and contralateral forepaws strength and motility were scored by two experimentally blinded evaluators using the following forelimb akinesia scale. In a scale of 1 to 3, 1 = normal, 2 = impaired and 3 = severely impaired. Scores were tallied for each individual animal, then mean scores for treatment groups were used for analyses.

### Paw Grasps Test

Pre- and post stroke surgery, grip strength of animals from stroke-vehicle, and stroke-EPC were evaluated. An abnormal grip is indicative of impaired neuromuscular function. In this test rats were held by their bodies against a pole [42]. Both ipsilateral and contralateral paw grip strength were scored by two experimentally blinded evaluators using the following grip strength scale. In a scale of 1 to 3, 1 = normal, 2 = impaired and 3 = severely impaired. Scores were tallied for each individual animal, then mean scores for treatment groups were used for analyses.

### Rotarod Test

Rotarod test was also performed to evaluate the degree of hemiparesis and coordinated movements. Prior to MCAo surgery, all rats were pre-trained to stay on the accelerating rotarod (Accuscan, USA) at a constant speed of 10 rpm until they could remain on the rotarod for 60 s. After 1 day of pre-training, we performed three trials in which the rotational speed was gradually increased from 5 to 20 rpm within 10 s. The longest time the rats remained on the rotarod was measured as the baseline. Thereafter, the data at day 1, 7, and day 30 post-MCAo are presented as averaged time on the rotarod of three trials (for each time point) relative to the baseline [3].

## Brain and Organ Harvesting, Fixation, and Sectioning

Under deep anesthesia, rats were euthanized on Day 7 post transplantation for immunofluorescent and protein analysis. For immunofluorescent analysis, briefly, animals were perfused through the ascending aorta with 200 mL of cold PBS, followed by 200 mL of 4% paraformaldehyde in phosphate buffer (PB). Brains were harvested and post-fixed in the same fixative for 72 h, followed by 30% sucrose in PB until completely sunk. Series of coronal sections were cut at a thickness of 40 μm using a cryostat and stored at −20 °C. For western blot protein analysis, briefly, animals were perfused through the ascending aorta with 200 mL of cold PBS. Brains were harvested and coronal sections were collected at a thickness of 2 mm using a brain matrix. The whole ipsilateral cortex and striatum were collected, homogenized and stored at −80 °C.

## Immunofluorescent

### Measurement of Cell Survival: Human Nuclei (HuNu) Staining Analysis

Every sixth 40 μm thick coronal tissue sections of brain, spanning the area of injury were randomly selected for quantitative analysis. Free floating sections were washed three times for 5 minutes in 0.1 M of phosphate buffered saline (PBS). For HuNu staining, samples were blocked for 60 min at room temperature with 5% normal goat serum (Invitrogen, CA) in PBS containing 0.1% Tween 20(PBST) (Sigma). Sections were then incubated overnight at 4 °C with mouse monoclonal anti-HuNu (1:50; Millipore, MAB1281) with 5% normal goat serum. The sections were washed five times for 10 minutes in PBST and then soaked in 5% normal goat serum in PBST containing corresponding secondary antibodies, goat anti-mouse IgG-Alexa 488 (green) (1:500; Invitrogen), for 90 min. Finally, sections were washed five times for 10 minutes in PBST and three times for 5 minutes in PBS, then processed for Hoechst 33258 (1:300; bisBenzimideH 33,258 trihydrochloride, Sigma) for 30 min, washed in PBS, and cover-slipped with Fluoromount (Sigma). Brain sections were examined using a confocal microscope (Zeiss). Control studies included exclusion of primary antibody substituted with 5% normal goat serum in PBS. No immunoreactivity was observed in these controls.

### Vasculome Immunofluorescent Staining

Inflammation-associated stroke vasculome including Brahma (BRM), IκB (also called NFκB inhibitor), Foxf1, and ITIH-5 stainings were carried out in the peri-infarct area of the cortex and striatum. All inflammation-associated vasculome species were conducted on every 1/3 sections, 40 μm thick, coronal brain sections. Briefly, brain sections were washed three times for 10 min in 0.1 M tris buffer solution (TBS). Six sections were incubated with saline sodium citrate (SSC) solution at PH 6.0 for 40 min at 80 °C for antigen retrieval purposes. Then, samples were blocked for 60 min at room temperature with 8% normal goat serum (Invitrogen, CA) in 0.1 M TBS containing 0.1% Tween 20 (TBST) (Sigma). Sections were then incubated overnight at 4 °C with rabbit polyclonal anti-BRM (1:100; ab23194; Abcam), rabbit polyclonal anti-Foxf1 (1:100; ab23194; Abcam), rabbit polyclonal anti-p-IkB (1:400; sc-8404; Santa Cruz) or mouse monoclonal anti-ITI-H5 (1:500; sa-398,538; Santa Cruz) with 10% normal goat serum. Then, the sections were washed five times for 10 minutes in 0.1 M TBST and soaked in 5% normal goat serum in 0.1 M TBST containing corresponding secondary antibodies, goat anti-mouse igG-Alexa 488 (green) (1:400; Invitrogen) or goat anti-rabbit IgG-Alexa 594 (red) (1:2000; Invitrogen) for 90 min. Finally, coronal sections were washed five times for 10 minutes in 0.1 M TBST and three times for 5 minutes in 0.1 M TBS, processed for 1:300 Hoechst 33258 (bisBenzimideH 33,258 trihydrochloride, Sigma) for 30 min, washed in 0.1 M TBS, and cover-slipped with Fluoromount (Aqueous Mounting Medium; sigma F4680). Coronal sections were examined using a confocal microscope (Zeiss). Control studies included exclusion of primary antibody substituted with 5% normal goat serum in 0.1 M TBS. No immunoreactivity was observed in these controls.

### Endothelial Cells Marker (RECA1) and Inflammation-Associated Stroke Vasculome Double Immunofluorescent Staining

Staining for specific rat endothelial cells (RECA1) and inflammation-associated stroke vasculome was done on every 1/3 coronal section throughout the entire peri-infarct area of the cortex and striatum for a total of 6 sections per staining. Briefly, brain sections were washed three times for 10 min in 0.1 M TBS. Six sections were incubated with saline sodium citrate (SSC) solution at PH 6.0 for 40 min at 80 °C for antigen retrieval. Then, samples were blocked for 60 min at room temperature with 8% normal goat serum (Invitrogen, CA) in 0.1 M TBS containing 0.1% Tween 20 (TBST) (Sigma). Sections were then incubated overnight at 4 °C with mouse anti-RECA1 (1:100; NC233393; AbD Serotec) with either rabbit polyclonal anti-BRM (1:100; ab23194; Abcam), rabbit polyclonal anti-Foxf1 (1:100; ab23194; Abcam), rabbit polyclonal anti-IkB (1:400; sc-945; Santa Cruz) or mouse monoclonal anti-ITI-H5 (1:500; sa-398,538; Santa Cruz) with 10% normal goat serum. Then, the sections were washed five times for 10 minutes in 0.1 M TBST and soaked in 5% normal goat serum in 0.1 M TBST containing corresponding secondary antibody, goat anti-mouse igG-Alexa 488 (green) (1:400; Invitrogen) and goat anti-rabbit IgG-Alexa 596 (red) (1:200; Invitrogen) for 90 min. Finally, coronal sections were washed five times for 10 minutes in 0.1 M TBST and three times for 5 minutes in 0.1 M TBS, processed for Hoechst (1:300; 33,258; Sigma) for 30 min, washed in 0.1 M TBS, and cover-slipped with Fluoromount (Aqueous Mounting Medium; sigma F4680). Coronal sections were examined using a confocal microscope (Zeiss). Control studies included exclusion of primary antibody substituted with 5% normal goat serum in 0.1 M TBS. No immunoreactivity was observed in these controls.

### Major Histocompatibility Complex II (MHCII) Staining

Staining for active microglia/macrophages antigen presenting cells MHCII (OX6) was done on every 1/6 coronal section throughout the entire peri-infarct area of the cortex and striatum for a total of 6 sections per staining. Briefly, brain sections were washed three times for 10 min in 0.1 M TBS. Six sections were incubated with saline sodium citrate (SSC) solution at PH 6.0 for 40 min at 80 °C for antigen retrieval purposes. Then, samples were blocked for 60 min at room temperature with 8% normal goat serum (Invitrogen, CA) in 0.1 M TBS containing 0.1% Tween 20 (TBST) (Sigma). Sections were then incubated overnight at 4 °C with mouse anti rat MHCII (1:750; 554,926; BD). Then, the sections were washed five times for ten minutes in 0.1 M TBST and soaked in 5% normal goat serum in 0.1 M TBST containing corresponding secondary antibody, goat anti-mouse igG-Alexa 488 (green) (1:400; Invitrogen) for 90 min. Finally, coronal sections were washed five times for 10 minutes in 0.1 M TBST and three times for 5 minutes in 0.1 M TBS, processed for 1:300 Hoechst 33258 (bisBenzimideH 33,258 trihydrochloride, Sigma) for 30 min, washed in 0.1 M TBS, and cover-slipped with Fluoromount (Aqueous Mounting Medium; sigma F4680). Coronal sections were examined using a confocal microscope (Zeiss). Control studies included exclusion of primary antibody substituted with 5% normal goat serum in 0.1 M TBS. No immunoreactivity was observed in these controls.

### Analysis of Fluorescent Staining

From all sections, approximately 4 to 6 images of 20X magnification were taken from each coronal section using confocal microscopy (Zeiss) and analyzed with ImageJ (National Institutes of Health, Bethesda, MD). All photomicrographs were converted to gray scale. Background was selected from blank control images, and subsequently used to subtract the background from all images. The same threshold was used for all images. Thereafter, the staining intensity of each section was quantified as the average optical density readings of 4 to 6 randomly selected areas within that section. The final staining intensity of each group resulted as the average of each staining intensity per section.

### Western Blot

The whole ipsilateral cortex and striatum were treated with RIPA lysis buffer (0.5 M Tris-HCl, pH 7.4, 1.5 M NaCl, 2.5% deoxycholic acid, 10% NP-40, 10 mM EDTA; 20–188; Sigma.) with protease inhibitor cocktail (I3786, Sigma-Aldrich). The lysate was centrifuged at 3000 g, 4 °C for 15 min, and the supernatant was stored at −80 °C until analysis. Protein samples (4~35 μg/lane) were processed on 4~14% Tris-Glycine SDS-PAGE gel and then transferred onto a nitrocellulose membrane (162–0112, Bio-Rad, Hercules, CA, USA) at 30 V, 4 °C for 14 h. The nitrocellulose membranes were treated with PBS containing 0.1% Tween-20 and 3% non-fat milk (170–6404, Bio-Rad) for 45 min at RT. Membranes were then incubated with the primary antibodies, rabbit polyclonal anti-BRM (1:100; ab23194; Abcam), rabbit polyclonal anti-Foxf1 (1:100; ab23194; Abcam), rabbit polyclonal anti-IkB (1:400; sc-945; Santa Cruz) or mouse monoclonal anti-ITI-H5 (1:500; sa-398,538; Santa Cruz) and anti-GAPDH mouse antibody (1:10,000; ab8245; Abcam) at 4 °C for 14 h. After washing with PBS containing 0.1% Tween-20 (PBST), the nitrocellulose membrane was incubated with donkey anti-mouse IRDye800®CW secondary antibody (1:5000; 926–32,212; LI-COR), or donkey anti-rabbit IRDye800®CW secondary antibody (1:5000; 926–32,213; LI-COR), for 90 min at RT in the dark. Immunoreactive detection using near-infrared fluorescence was performed according to the protocol of Odyssey® Infrared Imaging System (LI-COR®).

### Statistical Analysis

For behavioral data analyses, two-way ANOVA was used to evaluate statistical differences between treatment groups, followed by subsequent pairwise comparisons; post hoc tests Bonferroni’s test. For data analyses, ipsilateral corresponding brain areas were used as raw data providing 1 set of data per treatment condition (stroke-vehicle vs. stroke-EPC). Student’s t-tests were used to determine and compare the effect of stroke-vehicle versus stroke-EPC. All data are represented as mean values with ± SEM. Statistical significance was set at *p* < 0.05 for all analyses.

## Results

### EPC Treatment Downregulates the OGD-Induced Upregulation of Inflammation-Associated Stroke Vasculome In Vitro in HEN6 Cells

**Fig. 2:**
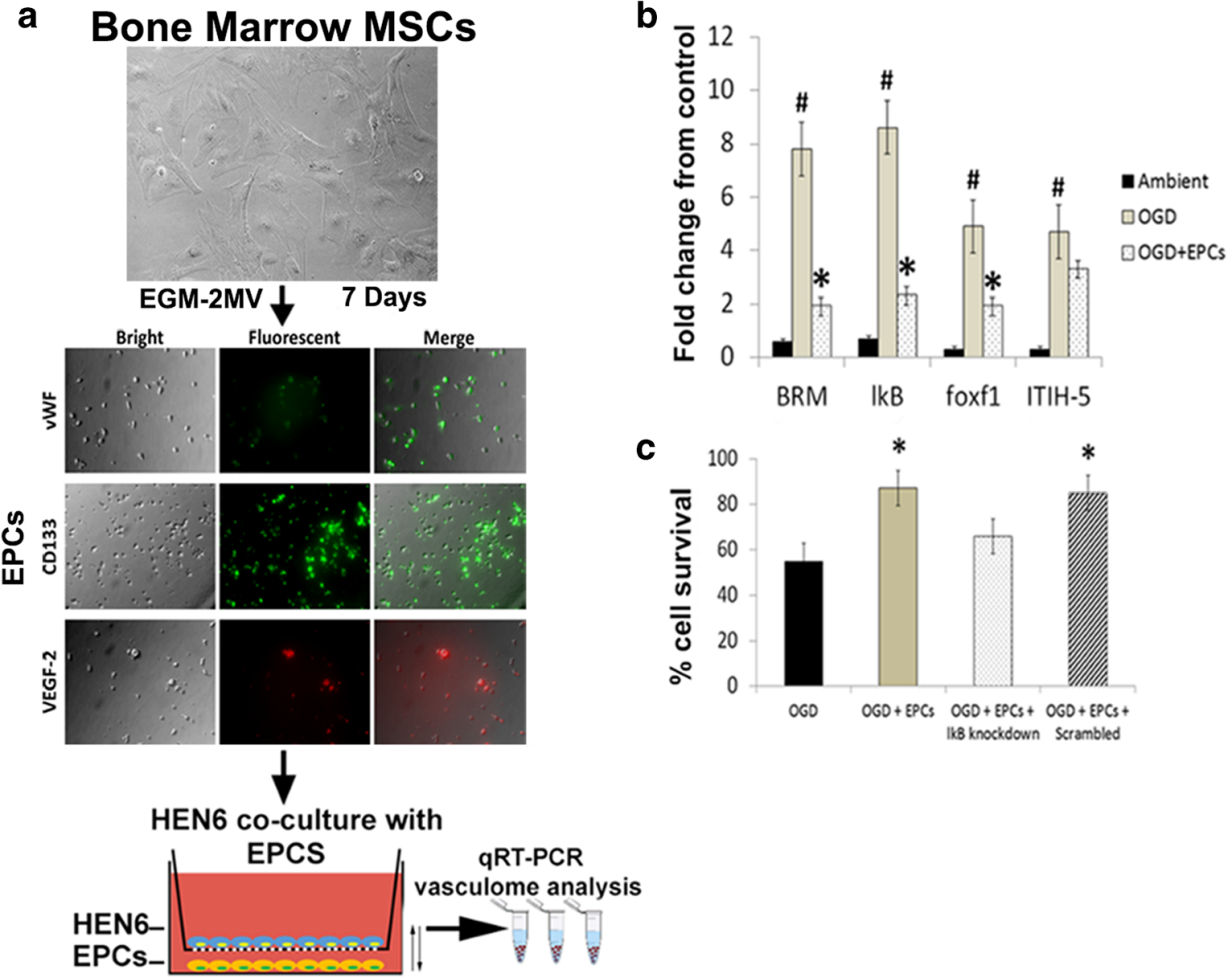
EPC treatment downregulates the OGD-induced upregulation of inflammation-associated stroke vasculome in vitro in HEN6 cells and increases cell viability. **a** Representative photomicrographs of human bone marrow mesenchymal stem cells (BM-MSCs) seeded with EGM-2MV medium supplemented with growth factors on collagen-I coated 48 well plates and incubated for 7 days. After 7 days, immunocytochemistry analysis revealed specific expression of EPC markers including vWF, CD133 and VEGFR-2. Next, human endothelial brain cells (HEN6) were subjected to oxygen and glucose degradation (OGD) and co-cultured with EPCs and levels of RNA vasculome were analyzed with qRT-PCR. **b** Quantitative analyses of total fold change of RNA vasculome revealed that BRM, IκB, Foxf1, and ITIH-5 levels increased after OGD up to 10 folds relative to ambient conditions (Two way ANOVA, F_3,21_ = 19.86, *p* < 0.001, Bonferonni’s test, *p* < 0.05). After EPC treatment, there was a significant 4, 6, and 2 fold decrease expression of BRM, IkB, and Foxf1 respectively in OGD-EPC treated HEN6 cells relative to OGD-vehicle treated HEN6 cells (p’s < 0.05), except ITI-H5 (*p* > 0.05). **c** Cell viability analysis showed there was a significant increase of HEN6 cell survival in OGD-EPC and OGD-EPC scramble treatment compared to OGD alone or OGD-EPC IkB knockdown (One way ANOVA, F_1,22_ = 44.58, *p* < 0.001, Bonferonni’s test, *p* < 0.05)

Using qRT-PCR technology, two-way ANOVA analysis demonstrated significant treatment effects (F_3,21_ = 19.86, *p* < 0.001). post hoc analysis revealed that under ambient condition, basal levels of BRM, IκB, Foxf1, and ITIH-5 could be detected in cultured human cerebral endothelial cells (HEN6) compared to non-endothelial human neuronal cell NT2N acting as control. Further analysis revealed, a significant upregulation of the inflammation-associated stroke vasculome including BRM, IκB, Foxf1, and ITIH-5 up to 10 folds after OGD relative to ambient conditions (p’s < 0.05). Co-culture with human EPCs (1:1 ratio) during OGD treatment demonstrated a significant 4, 6, and 2 fold decrease expression of BRM, IkB, and Foxf1 respectively in OGD-EPC condition relative to OGD-vehicle (p’s < 0.05), except ITI-H5 (*p* > 0.05). (Fig. 2a, b).

### Knockdown of IkB Block the EPC-Mediated Protection of HEN6 Against OGD

In order to decipher the therapeutic mechanism underlying the observed therapeutic benefits of EPCs after OGD treatment, an additional in vitro experiment using knockdown technology. ANOVA revealed significant treatment effects on cell survival post IKB knockdown after OGD and EPC treatment (F_1,22_ = 44.58, *p* < 0.001). Post hoc analysis revealed that after silencing the inflammation-associated stroke vasculome gene, IκB, as opposed to scrambled knockdown, significantly blocked the EPC-mediated protection of HEN6 against OGD as depicted by a significant decrease in cell survival in vitro (*p* < 0.05) (Fig. 2c).

### Transplantation of EPCs Improve Stroke-Associated Motor and Neurological Impairments in Stroke Rats

To test the hypothesis that administration of EPC grafts can rescue motor impairments associated with stroke neuropathology, a battery of motor behavioral, and neurological tests including EBST, forelimb akinesia, paw grasp and rotarod tests were conducted.

### EBST

**Fig. 3:**
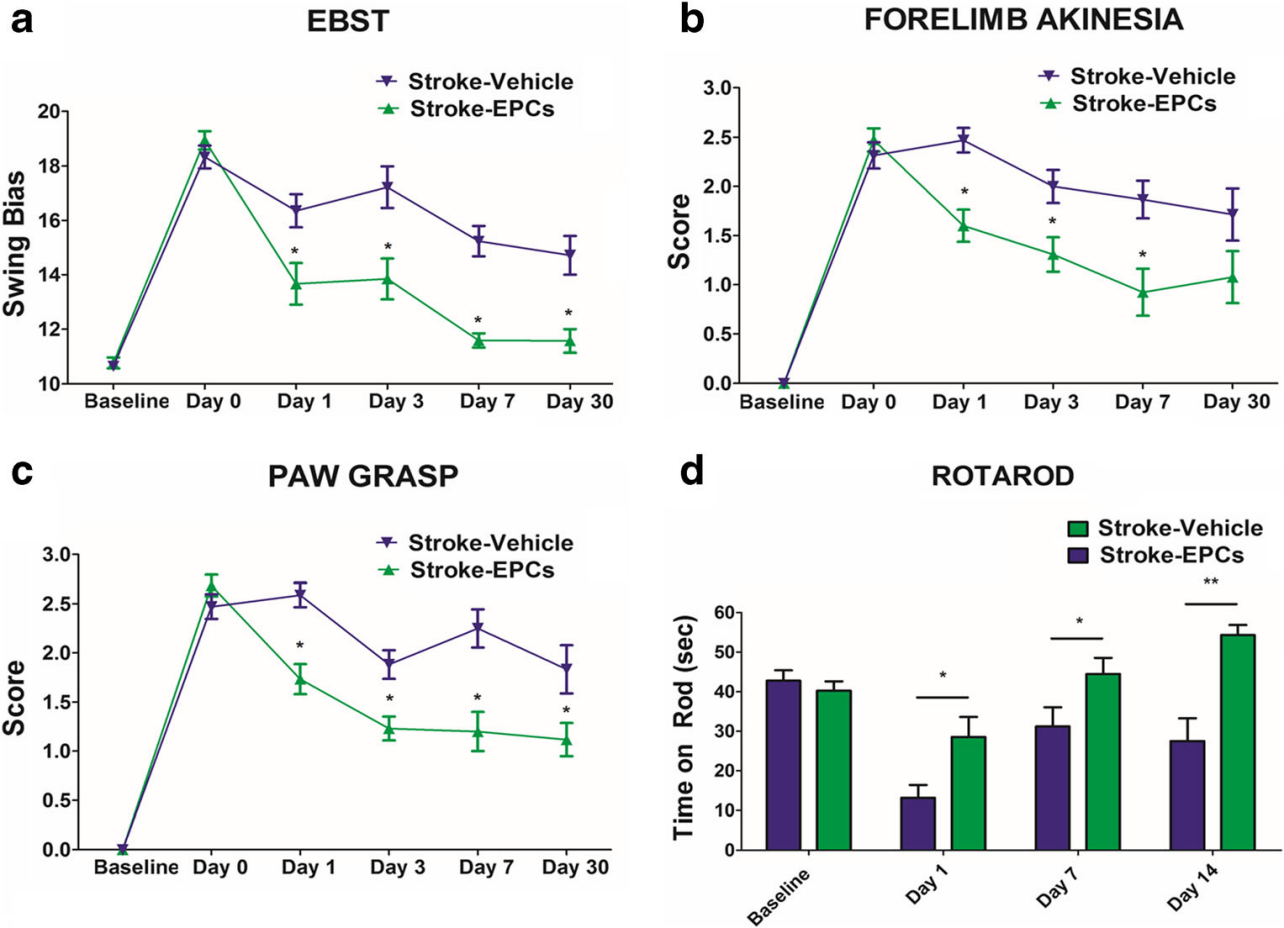
EPC transplantation ameliorates the stroke-associated motor and neurological impairments. Motor and neurological functions were assessed at baseline, post stroke, and post EPC transplantation on Day 0, 1, 3, 7, and 30. ANOVA revealed significant treatment effects in motor and neurological tests (EBST, F_1,22_ = 53.42, *p* < 0.0001, Bonferonni’s test *p* < 0.001; Forelimb akinesia, F_1,22_ = 65.60, *p* < 0.0001, Bonferonni’s test *p* < 0.01; Paw grasp test, F_1,22_ = 81.65, *p* < 0.0001, Bonferonni’s test *p* < 0.001; Rotarod task, F_1,22_  =  19.79, *p* < 0.0001, Bonferonni’s test *p* < 0.001). Pairwise analyses revealed that stroke-induced deficits, as seen in elevated body swing test (EBST), forelimb akinesia, paw grasp, and Rotarod, were reversed after treatment with EPCs relative to stroke-vehicle animals (*p* < 0.01)

ANOVA revealed significant treatment effects on EBST performance (F_1,22_ = 53.42, *p* < 0.0001). Post hoc test revealed a significant asymmetry in motor activity in animals subjected to stroke at day 0, 1, 2, 7 and 30 post-stroke relative to stroke-EPC animals (*p* < 0.001). Significant recovery of motor symmetry was detected as early as 24 h post treatment in stroke-EPC animals relative to stroke-vehicle animals and continued throughout testing period of 30 days (*p* < 0.001) (Fig. 3a).

### Forelimb Akinesia

Two-way ANOVA revealed significant treatment effects on forelimb strength (F_1,22_ = 65.60, *p* < 0.0001). Post hoc test revealed a significant decreased in forelimb movement and function in animals subjected to stroke at day 0, 1, 3, 7 and 30 post-stroke relative to stroke-EPC animals (*p* < 0.001). Significant forelimb motor movement amelioration was detected as early as 24 h post treatment in stroke-EPC treated animals relative to stroke-vehicle animals and continued throughout testing period of 30 days (*p* < 0.001) (Fig. 3b).

### Paw Grasp

Two-way ANOVA revealed significant treatment effects on grasp function or grip strength (F_1,22_ = 81.65, *p* < 0.0001). Post hoc test revealed a significant impairments in grasping abilities at day 0, 1, 3, 7, 30 post-stroke relative to stroke-EPC animals (*p* < 0.001). Significant recovery of grip strength was detected as early as 24 h post treatment in stroke-EPC animals relative to stroke-vehicle animals and continued throughout testing period of 30 days (*p* < 0.001) (Fig. 3c).

### Degree of Hemiparesis and Coordination Measured with Rotarod Performance

Similarly, significant treatment effects were detected in motor balance and coordination as revealed by two-way ANOVA (F_1,22_  =  19.79, *p* < 0.001) in the Rotarod test. Rotarod testing conducted prior to MCAo showed there was no significant difference between the two groups of animals, in that all animals learned to balance on the rotating rod for about 60 s (*p* > 0.05) (Fig. 3d). Post hoc test revealed a significant decrease in the time spent balancing on the rotating rod in stroke-vehicle animals relative to stroke-EPC animals (*p* < 0.0001). There was a significant amelioration of the balance and coordination of stroke-EPC animals compared to stroke-vehicle animals on day 1, 7, and 30 (*p* < 0.0001) (Fig. 3d).

### Human EPCs Immunodetection in the Brain

**Fig. 4:**
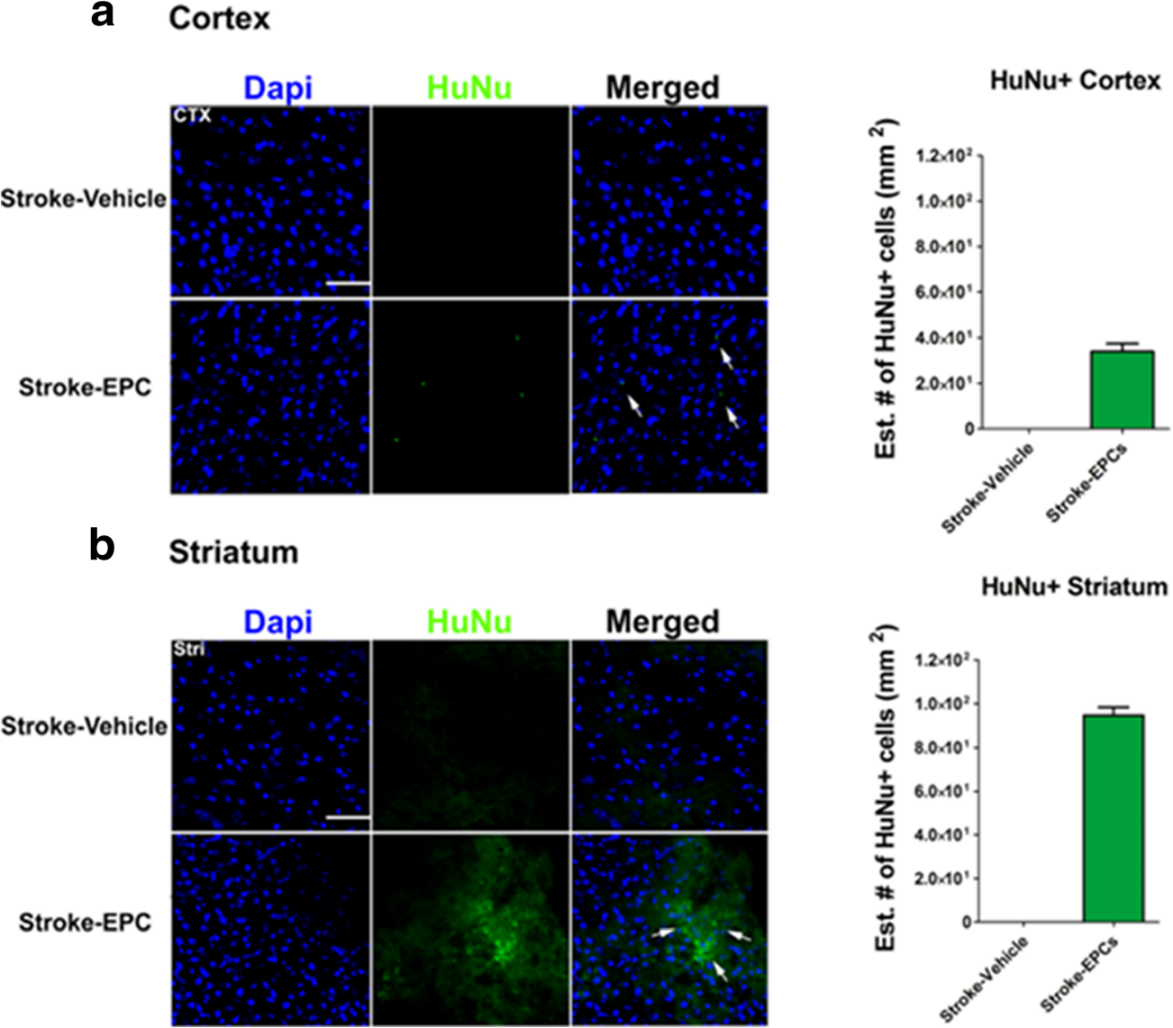
EPC engraftment in the cortex and striatum of transplanted stroke animals. **a** Representative merged images showed co-localization of HuNu+ and DAPI+ expression from grafted EPCs in the peri-infarct area of the cortex for the transplanted stroke-EPC animals as opposed to stroke-vehicle animals at 7 days post EPC transplantation. Top left, quantitative analyses of the estimated number of HuNu+ EPCs in the cortex revealed 0.04% or 40 EPCs in cortex peri-infarct area. **b** Representative merged images showed co-localization of HuNu+ and DAPI+ expression from grafted EPCs in the peri-infarct area of the striatum for the transplanted stroke-EPC animals as opposed to stroke-vehicle animals. Bottom left, quantitative analyses of the estimated number of HuNu+ EPCs in the striatum revealed 0.05% or 100 EPCs in cortex peri-infarct area. Scale bar: 50 μm. Data are expressed as mean ± SEM

Confocal microscopy using immunofluorescent demonstrated positive expression of transplanted EPCs in the brain of stroke-EPC transplanted stroke animals compared to stroke-vehicle animals in cortex (Fig. 4a) and in striatum (Fig. 4b). The mean graft survival of EPCs (i.e., HuNu expression) was estimated to be about 0.04% or 40 EPCs in peri-infarct areas in the cortex (Fig. 4a) and an estimated 0.05% or 100 EPCs in peri-infarct areas in the striatum (Fig. 4b).

### EPC Grafts Ameliorate Stroke–Induced Upregulation of Inflammation-Associated Stroke Vasculome in Cortex Peri-Infarct Area

**Fig. 5:**
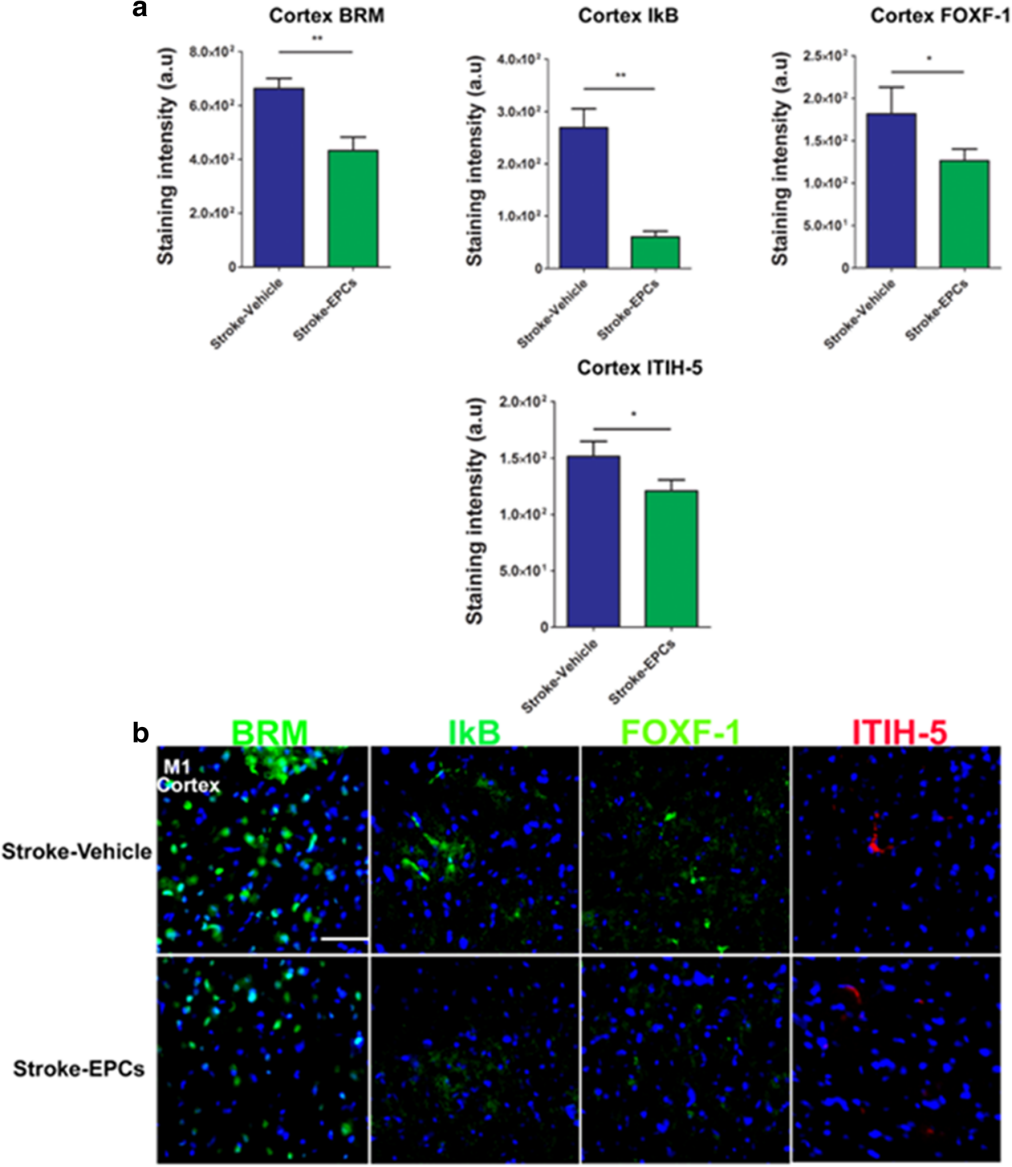
EPC transplantation decreases the inflammation-associated stroke vasculome at day 7 post-stroke in the cortex peri-infarct area. **a** Quantitative analysis of fluorescent intensity revealed significant downregulation of the inflammation-associated stroke vasculome BRM, IKB, Foxf1, and ITIH-5 in the ipsilateral M1 peri-infarct area of the cortex at 7 days post EPC transplantation (p’s < 0.01). **b** Photomicrographs correspond to the coronal sections of the ipsilateral hemisphere showing positive vasculome staining of the cortex M1 peri-infarct area in stroke-vehicle (top panel) and stroke-EPC (bottom) treated animals. Arrows indicate the area with positive immunostaining for BRM, IKB, Foxf1, and ITIH-5. Scale bar = 50 μm. Student t-test, p’s < 0.01. Data are expressed as mean ± SEM

Positive expression of inflammation-associated stroke vasculomes (BRM, IκB, Foxf1, and ITIH-5), were quantified using immunofluorescent techniques and analyzed with Image J software. There were significant downregulations of BRM, IκB, Foxf1, and ITIH-5 expression in the peri-infarct area of the cortex of stroke-EPC animals compared to stroke-vehicle animals at 7 days post EPC transplantation (Student t-test; p’s < 0.05) (Fig. 5a, b).

### EPCs Ameliorate Stroke–Induced Upregulation of Inflammation-Associated Stroke Vasculome in the Peri-Infarct Area of the Striatum

**Fig. 6:**
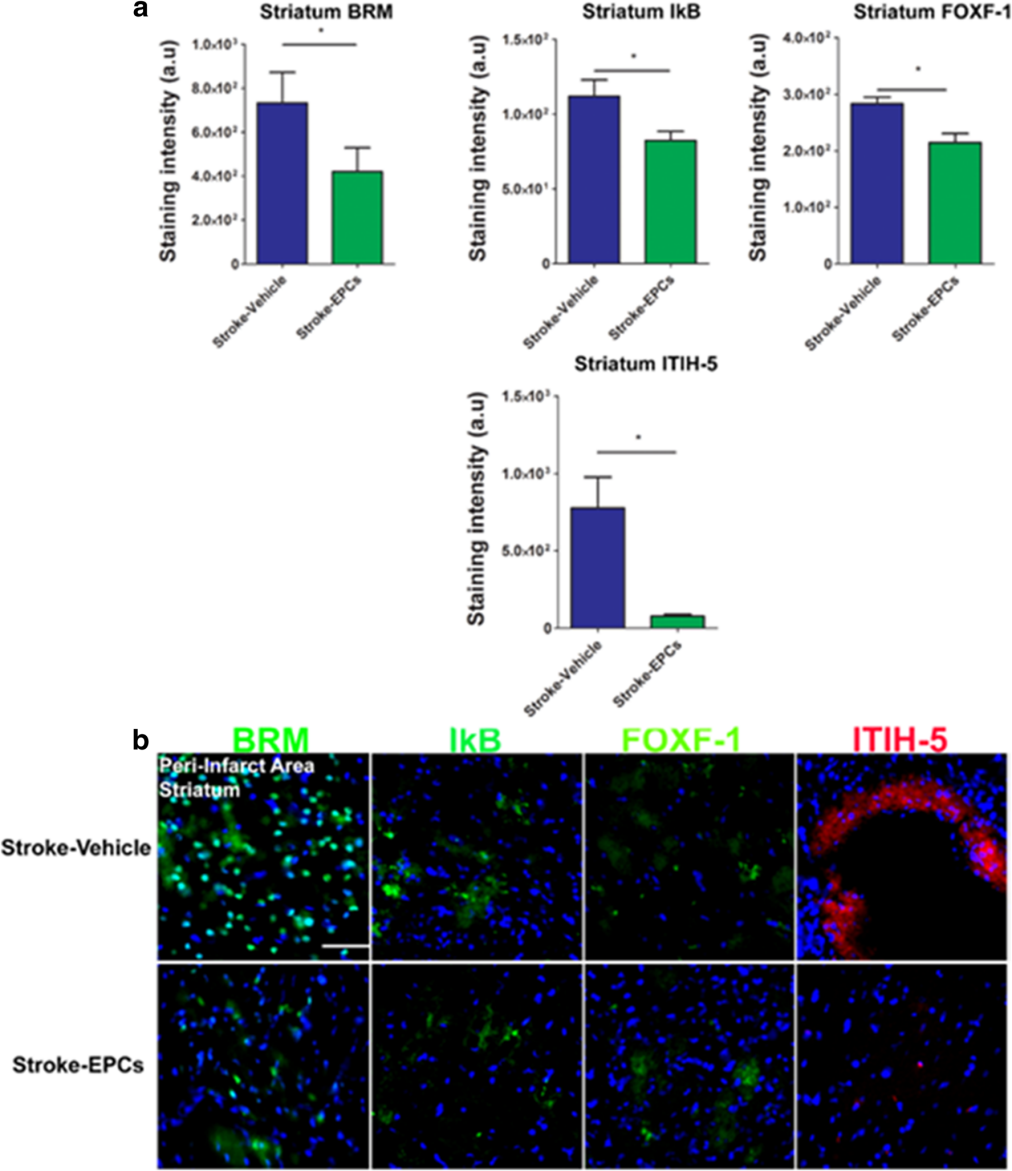
EPC transplantation decreases the inflammation-associated stroke vasculome at day 7 post-stroke in the striatum peri-infarct area. **a** Quantitative analysis of fluorescent intensity revealed significant downregulation of the inflammation-associated stroke vasculome BRM, IKB, Foxf1, and ITIH-5 in the ipsilateral peri-infarct area of the striatum at 7 days post EPC transplantation (p’s < 0.01). **b** Photomicrographs correspond to the coronal sections of the ipsilateral hemisphere showing positive vasculome staining of the striatum peri-infarct area in stroke-vehicle (top panel) and stroke-EPC (bottom) treated animals. Arrows indicate the area with positive immunostaining for BRM, IKB, Foxf1, and ITIH-5. Scale bar = 50 μm. Student t-test, p’s < 0.01. Data are expressed as mean ± SEM

Similarly, positive expression of inflammation-associated stroke vasculomes (BRM, IκB, Foxf1, and ITIH-5), were quantified using immunofluorescent techniques and analyzed with Image J software. There were significant downregulation of BRM, IκB, Foxf1and ITIH-5 expression in the peri-infarct area of the striatum of stroke-EPC animals compared to stroke-vehicle animals at 7 days post transplantation (Student t-test; p’s < 0.05) (Fig. 6a, b).

### EPC Grafts Decrease Vasculome/RECA-1 Ratio in the Peri-Infarct Area of the Cortex

**Fig. 7:**
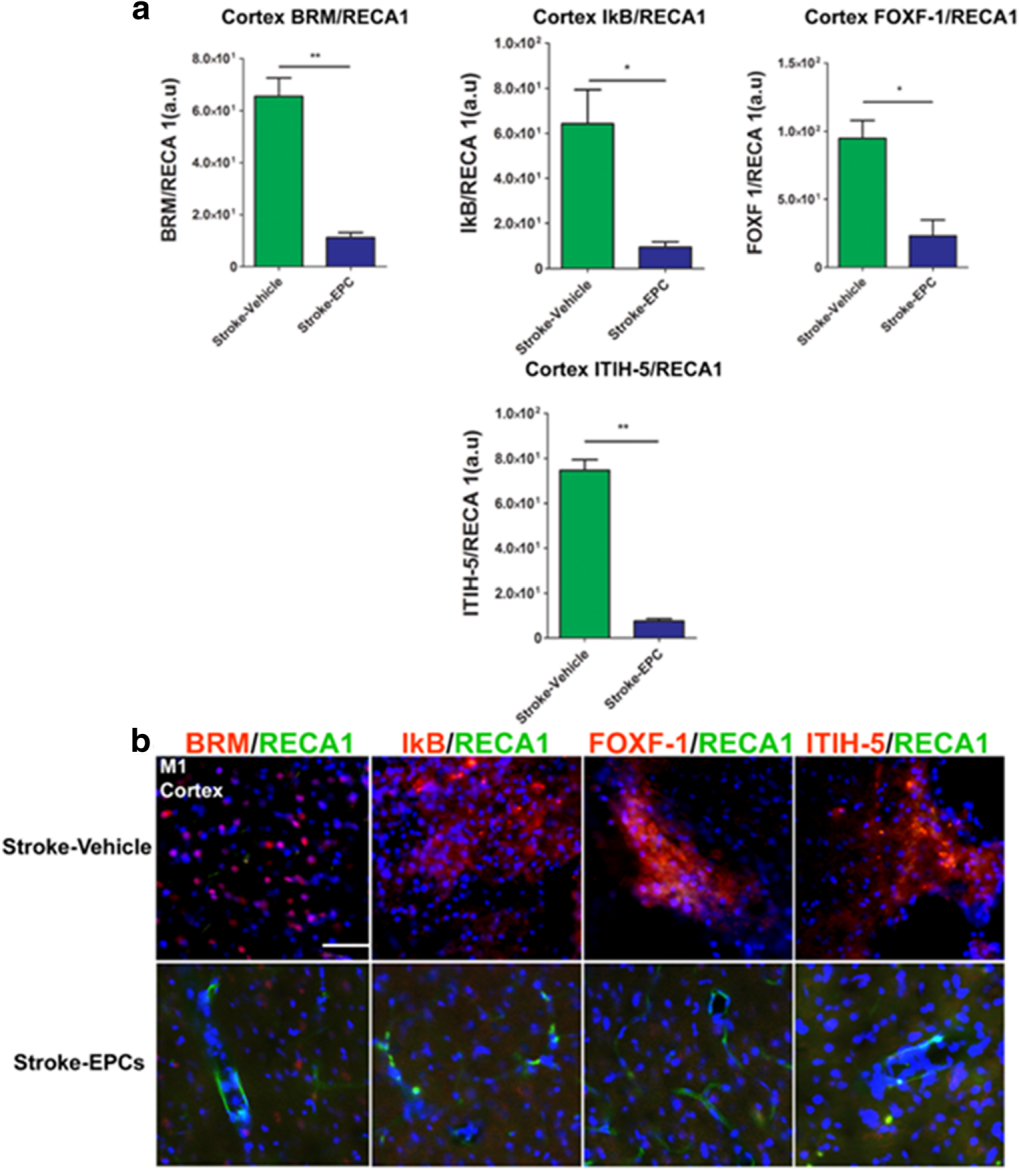
EPC transplantation decreases vasculome/RECA1 ratio at day 7 post-stroke in the cortex peri-infarct area. **a** Quantitative analysis of fluorescent intensity revealed significant reduction of the inflammation-associated stroke vasculome BRM, IKB, Foxf1, and ITIH-5 and blood vessel ratio in the ipsilateral peri-infarct area of the cortex at 7 days post EPC transplantation (p’s < 0.01). **b** Photomicrographs correspond to the coronal sections of the ipsilateral hemisphere showing positive vasculome and blood vessel positive RECA1 staining of the peri-infarct area of the cortex in stroke-vehicle and stroke-EPC treated animals. Arrows indicate co-localization of vasculome with RECA 1 staining for BRM, IKB, Foxf1, and ITIH-5, Scale bar = 50 μm. Student t-test, p’s < 0.01. Data are expressed as mean ± SEM

Quantitative analysis of co-localization of inflammation-associated stroke vasculome and brain blood vessels (RECA-1) fluorescent intensity demonstrated significant treatment effects on in the ipsilateral M1 region of the cortex as evidence by the increased ratio vasculome/ RECA-1 in stroke-vehicle animals relative to stroke-EPC animals (p’s < 0.01). The EPC treatment effect is reflected in the decreased vasculome/RECA 1 ratio whereby vasculome expressions are decreasing and RECA 1 is dominating. Vasculome/ RECA-1 ratio analysis revealed significant upregulation of the inflammation-associated stroke vasculome BRM, IKB, Foxf1, and ITIH-5 in the peri-infarct area of the cortex of stroke-vehicle animals at 7 days post EPC transplantation relative to the vasculome/ RECA-1 ration of the stroke-EPC animals (Student t-test; p’s < 0.01). (Fig. 7a, b).

### EPC Grafts Decrease Vasculome/RECA-1 Ratio in Striatum Peri-Infarct Area

**Fig. 8:**
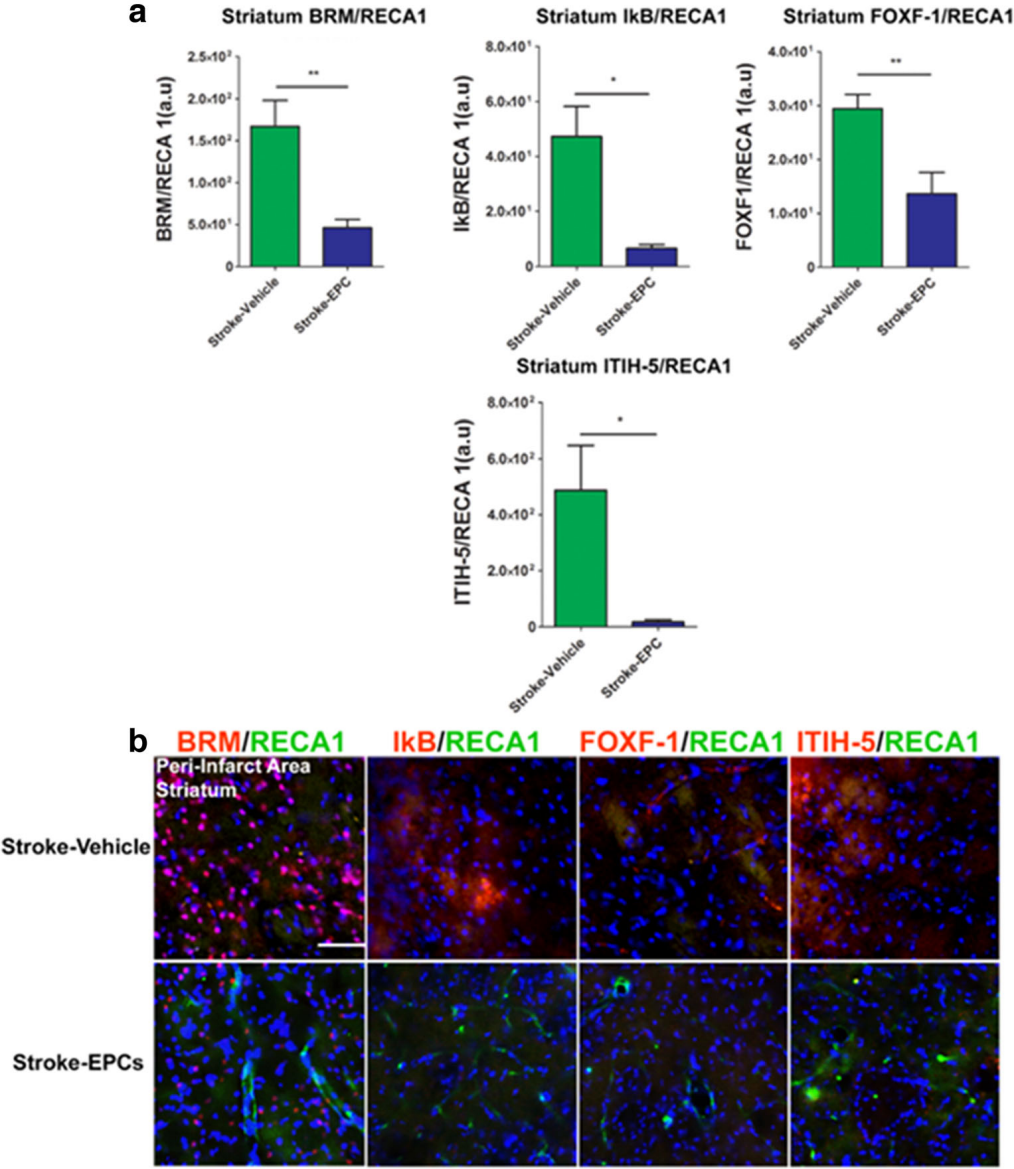
EPC transplantation decreases vasculome/RECA1 ratio at day 7 post-stroke in the striatum peri-infarct area. **a** Quantitative analysis of fluorescent intensity revealed significant reduction of the inflammation-associated stroke vasculome BRM, IKB, Foxf1, and ITIH-5 and brain blood vessel (RECA1) ratio in the ipsilateral peri-infarct area of the striatum at 7 days post EPC transplantation (p’s < 0.01). **b** Photomicrographs correspond to the coronal sections of the ipsilateral hemisphere showing positive vasculome and brain blood vessel positive (RECA1) expression in the peri-infarct area of the striatum in stroke-vehicle and stroke-EPC treated animals. Arrows indicate co-localization of vasculome with RECA 1 staining for either BRM, IKB, Foxf1, and ITIH-5, Scale bar = 50 μm. Student t-test, p’s < 0.01. Data are expressed as mean ± SEM

Quantitative analysis of the co-localization of inflammatory-associated stroke vasculome and brain vessels (RECA-1) fluorescent intensity demonstrated significant treatment effects in the peri-infarct region of the striatum as evidence by the increased ratio vasculome/RECA-1 in stroke-vehicle animals relative to stroke-EPC animals (Student t-test; p’s < 0.01). Vasculome/RECA-1 ratio analysis revealed significant upregulation of the inflammation-associated stroke vasculome BRM, IKB, Foxf1, and ITIH-5 in the striatum peri-infarct area of stroke-vehicle animals 7 days post transplantation relative to the vasculome/RECA-1 ration of the stroke-EPC animals (Student t-test; p’s < 0.01). (Fig. 8a, b).

### Overexpression of MHCII+ Cells Is Attenuated Post EPC Transplantation in the Peri-Infart Area of the Cortex in Stroke Animals

**Fig. 9:**
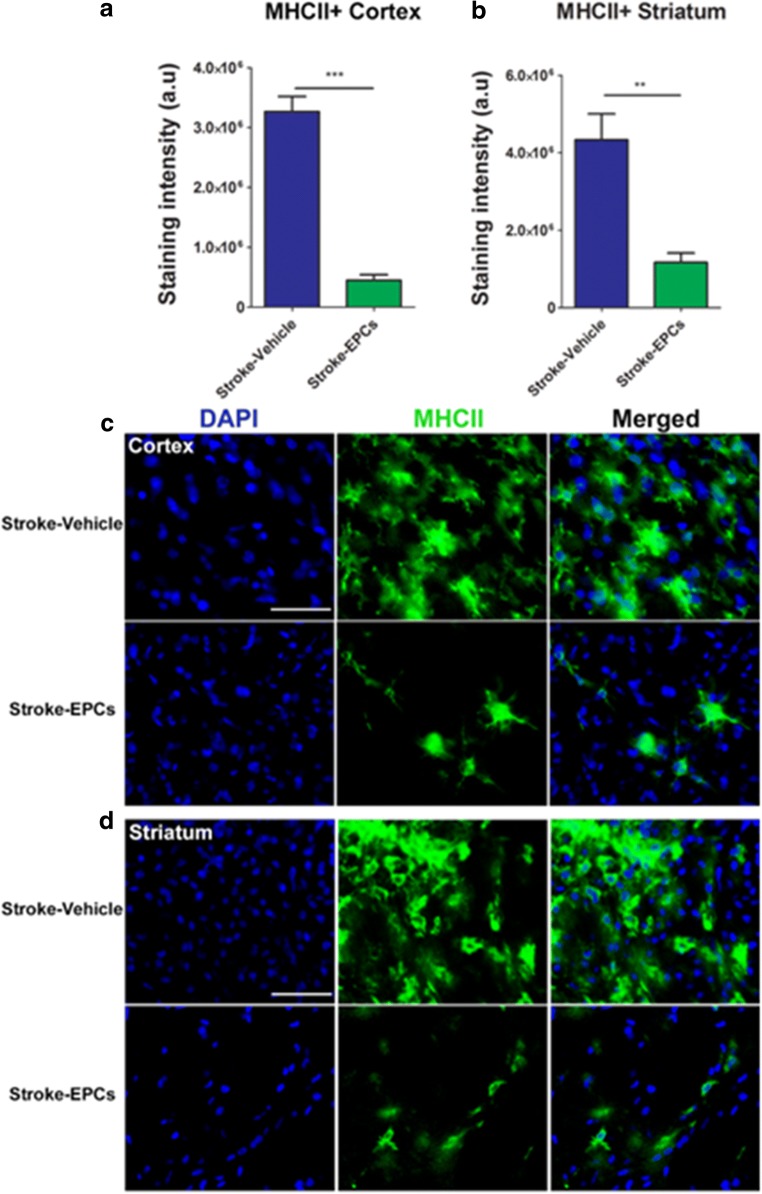
EPC transplantation reduces neuroinflammation in peri-infarct area at 7 days post- stroke. **a** and **b**, Quantitative analysis of the staining intensity of MHCII+ in the cortex and striatum revealed significant downregulation of activated MHCII+ cells in the stroke-EPC transplanted animals relative to stroke-vehicle animals across the brain (p’s < 0.001), except in SVZ (*p* > 0.05). EPC transplantation promoted a 78%, and 58% reduction of the estimated expression of MHC+ cells in the ipsilateral cortex and striatum, respectively relative to the ipsilateral side of the same regions of stroke-vehicle animals (p’s < 0.001). **c** and **d** Representative photomicrographs of coronal brain sections ipsilateral to injury immunostained with MHCII at 7 days post EPC transplantation. Arrows indicate positive staining for activated MHCII+ cells in the cortex (**c**) and striatum (**d**). Scale bar = 50 μm. Student t-test, *p* < 0.001. Data are expressed as mean ± SEM

Positive expression of exacerbated activated microglia/macrophages (MHCII+) was quantified in the cortex using immunofluorescent techniques and analyzed with Image J software. There was a significant downregulation of MHCII+ cells in the peri-infarct area of the cortex of stroke-EPC animals compared to stroke-vehicle animals 7 days post transplantation (Student t-test; p’s < 0.05) (Fig. 9a, c).

### Overexpression of MHCII+ Cells Is Attenuated Post EPC Transplantation in Peri-Infarct Area of the Striatum in Stroke Animals

Positive expression of exacerbated activated microglia/macrophages (MHCII+) was quantified in striatum using immunofluorescent techniques and analyzed with Image J software. There was a significant downregulation of MHCII+ cells in the peri-infarct area of the striatum of stroke-EPC animals compared to stroke-vehicle animals 7 days post transplantation (Student t-test; p’s < 0.05) (Fig. 9b, d).

### Validation of Inflammation-Associated Stroke Vasculomes Protein Levels in Ipsilateral Cortex Lysates 7 Days Post EPC Transplantation

**Fig. 10:**
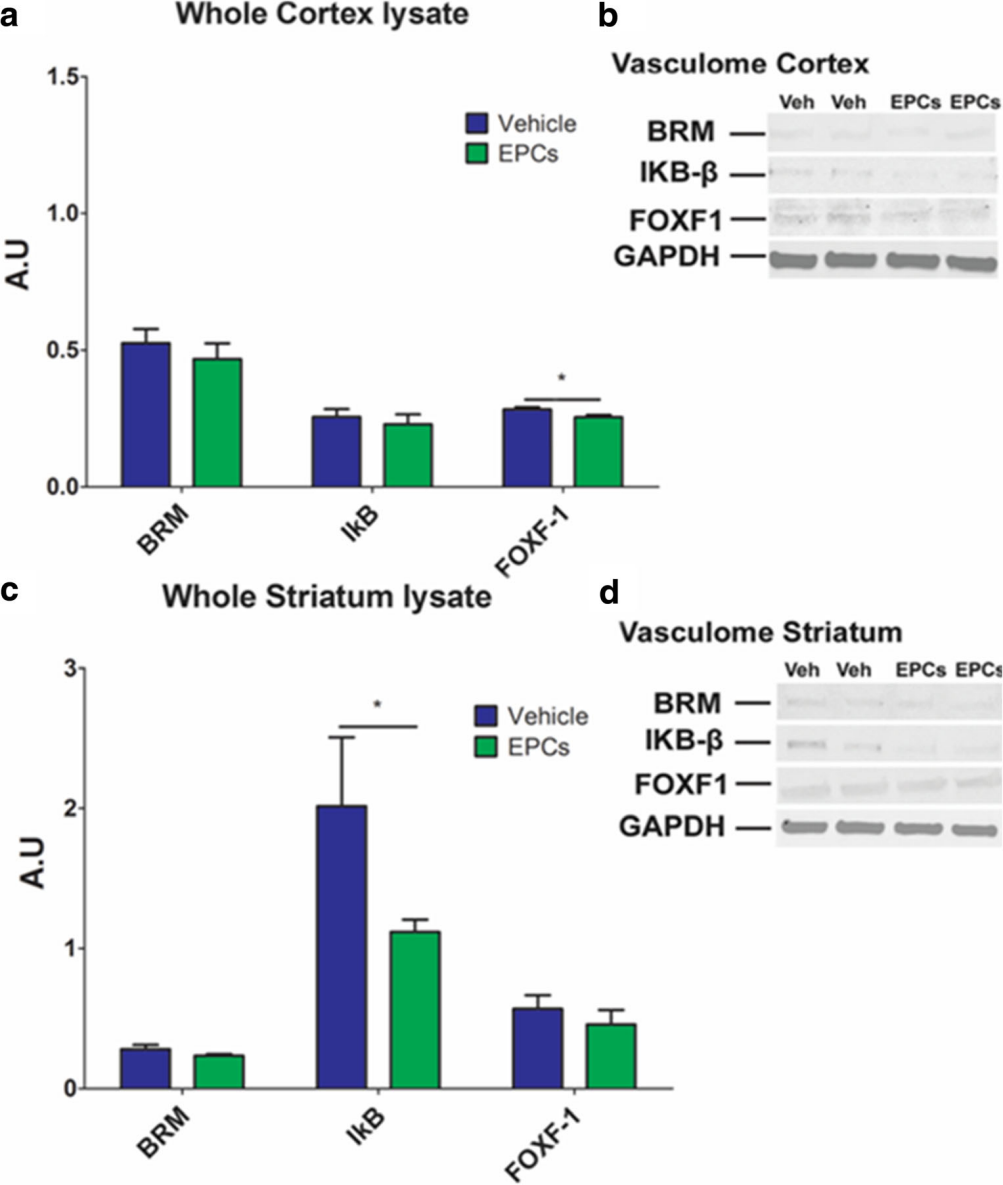
EPC transplantation decreases Foxf1 protein levels in the whole ipsilateral cortex and IkB in the whole ipsilateral striatum at 7 days post transplantation. **a** Relative quantification of protein expression levels (a.u) revealed a significant decrease of protein expression of Foxf1 in the whole ipsilateral cortex lysate of stroke-EPC transplanted animals relative to stroke-vehicle animals (Two way ANOVA, F_1,22_ = 26.35, *p* < 0.0001). **b** Expression levels of inflammation-associated stroke vasculome BRM, IkB, and Foxf1 and GAPDH. **c** Similarly, relative quantification of protein expression levels (a.u) in whole ipsilateral striatum reveled a significant decrease of protein expression of IkB in the whole ipsilateral striatum lysate of stroke-EPC transplanted animals relative to stroke-vehicle animals (Two way ANOVA, F_1,22_ = 21.19, *p* < 0.0001). **d** Expression levels of inflammation-associated stroke vasculome BRM, IkB, and Foxf1 and GAPDH. Experiments were independently conducted 3~5 times

Western blot analysis demonstrated the protein expression of inflammation-associated stroke vasculomes BRM, IkB, and Foxf1 in ipsilateral cortex lysates 7 days post EPC transplantation. Expression analysis 7 days post EPC transplantation revealed a significant downregulation of Foxf1 protein expression of stroke-EPC animals compared to stroke-vehicle animals (Student t-test; *p* < 0.05) in the whole ipsilateral cortex lysates. There were not significant differences of protein levels of BRM and IkB of stroke-EPC animals relative to stroke-vehicle animals (Student t-test; p’s > 0.05) in the whole ipsilateral cortex lysates 7 days post EPC transplantation. ITI-H5 showed undetectable levels in the whole ipsilateral cortex lysates (Fig. 10a, b).

### Validation of Inflammation-Associated Stroke Vasculomes Protein Levels in Ipsilateral Striatum Lysates 7 Days Post EPC Transplantation

Western blot analysis demonstrated the protein expression of inflammation-associated stroke vasculomes BRM, IkB, and Foxf1 in whole ipsilateral striatum lysates 7 days post EPC transplantation. Expression analysis 7 days post EPC transplantation revealed a significant downregulation of IkB protein expression of stroke-EPC animals compared to stroke-vehicle animals (Student t-test; p’s < 0.05) in the whole ipsilateral striatum lysates. There was a trend towards significant decrease of BRM in the striatum of stroke-EPC animals relative to stroke-vehicle animals (*p* > 0.05). There were not significant differences of protein level of Foxf1 of stroke-EPC relative to stroke-vehicle animals (Student t-test; *p* > 0.05) in the whole ipsilateral striatum lysates 7 days post EPC transplantation. ITI-H5 showed undetectable levels in the whole ipsilateral striatum lysates (Fig. 10c, d).

## Discussion

In the present study, we provided evidence for modulation of inflammation-associated stroke vasculome by exogenous transplantation of EPCs during acute time points in vitro and in vivo. In vitro detailed analyses of the human endothelial cells (HEN6) during OGD indicated a massive upregulation of inflammation-associated stroke vasculome (BRM, IkB, Foxf1, ITI-H5) relative to ambient conditions. Further analysis revealed a significant downregulation of the stroke vasculome after co-culture with EPCs. Next, silencing the inflammation-associated stroke vasculome gene, active IκB, as opposed to scrambled knockdown, blocked the EPC-mediated protection of HEN6 against OGD. Furthermore, in vivo transplantation of EPCs reduced overexpression of the inflammation-associated stroke vasculome coupled with downregulation of the immune response, specifically MHCII positive cells in the peri-infarct area of cortex and striatum. Similarly, we found EPC transplantation mediated the amelioration of brain vessels in the peri-infarct area of stroke animals. Moreover, it was observed that EPC-transplantation significantly lessened the motor and neurological impairments associated with stroke pathology during acute time points. Altogether, these results suggest that the upregulated expression of inflammation-associated stroke vasculome may be linked to the onset and progression of neurodegeneration and neuroinflammation after the initial post stroke injury and that EPC transplantation is able to sequester this secondary cell death by modulating the overexpression of inflammation-associated stroke vasculome.

Previously, it has been shown that acute ischemic stroke is associated with an array of predominantly inflammation-associated gene response [20, 43–45]. The “sick” stroke genes associated with regulation and modulation of the inflammatory response after stroke include Brahma (BRM), NF-kb inhibitor kappa B alpha (IkB), Forkhead box F1 (Foxf1), and Inter-alpha-trypsin inhibitor (ITI-H5). This supports the idea that the brain vasculature is no longer regarded as an inert system with little or no interaction with the brain physiology and homeostasis. In fact, it has been shown that the main cellular components of the cerebral vasculature, in particular the cerebral endothelium, have endocrine and paracrine functions thereby constantly interacting with the brain. EPC transplantation may support healthy endothelial cells, which are capable of providing signaling molecules and trophic factors that control brain metabolism, promote cellular proliferation and regulation of immune responses, which contribute with overall healthy brain function [20, 33, 46]. A closer analysis of these stroke vasculome showed that acute upregulation of mixed genes are important for blood pressure, vascular contraction, differentiation, tumorigenesis, embryo development, angiogenesis and inflammation [20].

Brahma (BRM) belongs to one of two ATPase catalytic subunits of the switch/sucrose non-fermentable (SWI/SNF). BRM and Brahma/SWI2-related gene 1(BRG1) have been found to play a vital part in regulating the transcription of different genes, including acting as a tumor suppressor for various cancers [47]. BRG1 also reduced mRNA expression of acute neuroprotective proteins including MMP3, TIMP Metallopeptidase inhibitor 2, cyclooxygenase 2, and IL6, which collectively decrease inflammation [47]. Similarly, BRG1 increases transcription of pro-inflammatory genes in retinal ischemia [48]. It has been shown that upregulation of BRG1 level at the first and last TNF-alpha exons occurs as early as 2 hours post ischemic retinal and was sustained for 1 week [48]. In addition, knockdown of BRG1 reduces the mRNA expression of TNF-alpha and MCP-1 as well as the recruitment of Pol II to TNF-alpha and MCP-1 genes [48]. In line with these findings, the present study showed aberrant overexpression of BRM in the peri-infarct area adjacent to the infarcted area of the cortex and striatum, which was attenuated after EPC transplantation at Day 7.

Moreover, IKB-alpha is known to be the regulatory protein that acts to inhibit and inactivate the transcriptional activity of NF-kB, which is found to play critical roles in inflammation, immunity, cell proliferation, differentiation, and survival [49]. IKB-alpha constitutes its inhibitory effect onto NF-kB through binding tightly to NF-kB dimers, forming a complex, masking the nuclear localization signals (NLS) and keeping the NF-kB sequestered in the cytoplasm [50]. Due to stressors like ischemia or hypoxia, during the acute phase there IkB-alpha gets activated when phosphorylated by IkB kinase (IKK) causing their eventual degradation by proteasomes [51, 52]. The ischemia-induced increase of active IkB-alpha expression in the brain allows increased NF-kB expression and leads to neuroinflammatory responses, which can cause harmful post-stroke injury [53]. Various studies have attempted to determine how eliminating the upregulation of phosphorylated IkB-alpha and its subsequent degradation can affect post-ischemia recovery. Interestingly, several studies have shown that ischemic post conditioning, the use of flavonoids and stem cells therapy can reversed the phosphorylation and degradation levels of IkB-alpha caused by ischemia [51, 52, 54, 55]. These studies support our finding whereby EPC graphs were able to reduce the overexpression of active IkB-alpha, typically seen after ischemic injury, in the peri-infarct area of cortex and striatum as depicted by immunofluorescent and further validated by protein analysis of the whole ipsilateral hemisphere.

Forkhead box F1 (Foxf1) is a part of the forkhead family of transcription factors and is an early marker of endothelial differentiation, which play a vital transcriptional factor in endothelial cell development, vasculogenesis, and vessel formation [56–58]. Embryology studies revealed that Foxf1 is implicated to be an important transcription factor for vascular development. It has been shown that Foxf1 knockout (Foxf1−/−) mice embryos do not survive past E10, thus solidifying the notion that Foxf1 is crucial for proper embryonic development [56]. Foxf1 has recently been linked to be a major regulator of S1pr1 transcription and stabilization of VE-cadherin in endothelial junctions in both healthy and injury vasculature in an experimental model of lung injury [57]. However, Foxf1 aberrant expression has been linked to alteration in downstream pathways in lung fibroblasts in idiopathic pulmonary fibrosis (IPF) [58]. Normal expression of Foxf1 was able to repressed aberrant cell growth and expression of collagen-1 (COL1) and actin-related protein 2/3 complex, subunit 2 (ARPC2) in normal fibroblast. In IPF fibroblasts, overexpression of Foxf1 blunted COL1 and ARPC2 repression, and cell growth was not repressed [58]. Foxf1 has also shown to act as an upstream activator of Flk1 gene expression [59]. As a vital transcription factor, the overexpression of Foxf1 in the peri-infarct area of the cortex and striatum is indicative of alteration related with vasculogenesis, endothelial junctions and cell development. Acute transplantation of EPCs ameliorated the Foxf1 aberrant expression in the peri-infarct area of cortex and striatum. Interestingly, rat endothelial cell marker demonstrated EPC transplantation also rescued the downregulation of peri-infarct microvessels in both cortex and striatum. In support of these findings, other studies have documented the ability of EPCs to secrete angiogenic factors such as VEGF, FGF-b, which are able to interact with Foxf1 at the molecular level and significantly increase capillary density in the peri-infarct area.

ITIH-5 is a member of the inter-alpha-trypsin inhibitor family, which contains a non-variable light chain (bikunin) and a variable heavy chain (H5 in ITIH-5). This heavy chain-to-hyaluronan interaction is facilitated by Tumor necrosis factor-inducible gene 6 (TSG-6) and is currently the only known interaction of the ITI heavy chain [60], serving to cross-link molecules within the ECM and enhance ECM rigidity [61]. ITIH-family proteins have been revealed to modulate peripheral inflammatory responses in multiple diseases [62]. The formation of extracellular or cell surface ITIH-HA complexes promotes the adhesion of leukocytes [60]. It could be ascertained that expression of ITIH-5 within cerebral endothelium could promote the adhesion and extravasation of circulating leukocytes through the damaged cerebral vasculature, suggesting a potential detrimental role [20, 63]. Yet, treatment with human ITI for inflammatory syndromes such as endotoxin shock has been shown to confer therapeutic effects, indicating its potential anti-inflammatory roles [64]. ITI family proteins are generally regarded now as being potent anti-inflammatories [65]. Interestingly, in the present study, it was found that ITIH5 is upregulated in vitro and in vivo after ischemic insult. Based on the present literature, the upregulation of ITIH5 7 days post stroke could account as a mechanism to regulate the exacerbation of the inflammatory response in our model. This aberrant upregulation of ITIH5 was downregulated after EPC transplantation. In concurrence with the notion of being a modulator of peripheral inflammatory responses, in the present study, there was a significant downregulation of MHCII positive cells in the peri-infarct area of cortex and striatum.

Furthermore, in vivo, motor and neurological ameliorations were achieved by EPC transplantation as early as 24 h post treatment and continued throughout testing period of 30 days. Animals subjected to stroke and treated with EPCs exhibited a robust amelioration on hemiparesis, motor symmetry and coordination as well as forelimb movement and grip strength relative to animals subjected to stroke treated with vehicle. In accordance, previously, both pre-clinical and clinical studies suggest that EPCs predominantly contribute in re-endothelialization during the neovascularization of ischemic infarcted areas and that may accounts for the functional improvement seen in animal models post stroke [66–68]. EPCs are known to significantly secrete angiogenic and vasculogenic molecule including bFGF, VEGF and BNDF [69]. At the functional level, quantitative analysis revealed significant attenuations of forelimb akinesia and neurological behavior using mNSS scoring metrics in EPC-transplanted ischemic animals and ischemic diabetic animals [70–72].

Ischemic stroke injury is a very intricate and dynamic event, whereby massive amount of reactive oxygen species, ATP exhaustion, endothelial and neuronal swelling, and exacerbated immune cell response causes the activation of pathways disrupting permeability barriers between blood and brain instigating further neuronal cell death and progressive secondary neurodegeneration of neurons, and microvessels [73–75]. Hypoxic-ischemic environments are recognized as a major physiological insult that induces cell death, activating inflammatory pathways in the CNS, triggering blood brain barrier disruption by increasing inflammation-associated vasculome, and altering brain homeostasis, altogether instigating vascular permeability and vasogenic edema [20, 76]. In tandem, there is a cascade of inflammatory pathways upregulating pro-inflammatory cytokine production, all of which in turn activating various cell death signals for necrosis and programed cell death. Of note, recent studies have demonstrated the involvement of neurotransmitter signaling in the modulation of neuroinflammation [77]. Hence, further studies that investigate the role of the inflammation-associated vasculome and purinergic signaling in ischemic stroke is essential. Interestingly, endothelial cells are particularly susceptible to ischemic injury, as a result of large numbers of mitochondria in their cytoplasm [78], suggesting that repair of the endothelial cells represent a key therapeutic target for stroke. The present observation of vasculature-directed treatment via EPC therapy stands as a potent approach in abrogating the inflammation-associated secondary cell death of stroke.
